# BPaLM Regimen Against Drug-Resistant Tuberculosis in India: A Need of the Hour

**DOI:** 10.7759/cureus.68421

**Published:** 2024-09-01

**Authors:** Sankalp Yadav

**Affiliations:** 1 Medicine, Shri Madan Lal Khurana Chest Clinic, New Delhi, IND

**Keywords:** multidrug-resistant tuberculosis (mdr-tb), extensively drug-resistant tuberculosis (xdr-tb), tuberculosis, moxifloxacin, linezolid, pretomanid, bedaquiline, bpalm

## Abstract

India has a high burden of drug-resistant tuberculosis (DR-TB) cases. The management of this severe form of TB is associated with a number of issues like long treatment durations, high pill burden, and multiple adverse drug reactions. Efforts are on through various research studies and trials for finding solutions to the issues linked to the current drug regimens against drug-resistant tuberculosis. One such remarkable development is the introduction of bedaquiline, pretomanid, linezolid, and moxifloxacin (BPaLM)-based regimens to fight against two of the most severe forms of tuberculosis, i.e., multidrug- and extensively drug-resistant tuberculosis (MDR-TB and XDR-TB). This editorial throws light on this newer regimen and discusses the same in the Indian context.

## Editorial

Tuberculosis (TB) is a disease documented to affect mankind for centuries. It is a public health scare, and with the development of drug-resistant strains in *Mycobacterium tuberculosis*, management has increasingly become challenging. Furthermore, India has a high burden of drug-resistant TB (DR-TB) cases. There are several types of DR-TB: extensively drug-resistant (XDR-TB), isoniazid mono-resistant (H-Mono), rifampicin-resistant TB (RR-TB), multidrug-resistant (MDR-TB), and pre-extensively drug-resistant (pre-XDR-TB). *M. tuberculosis* complex strains that are resistant to at least isoniazid and rifampicin are the cause of MDR-TB. With 1.3 million cases of DR-TB in 2018, India has the second-highest global burden of MDR-TB. A percentage (2.8%) of new cases and 12% of those that have already received treatment have MDR-TB. The incidence of MDR-TB/RR-TB is 135,000 in India [1].

The management of these cases is associated with a number of issues. These issues were due to high pill burden, multiple adverse drug reactions, and longer treatment duration, which ultimately affected the treatment outcomes, the development of severe forms of drug resistance, and morbidity in a significant number of cases. To address these issues, the World Health Organization recommended a six-month treatment regimen for DR-TB patients in its update on treatment guidelines in December 2022, replacing the nine-month or longer 18-month regimens. The new medication pretomanid, developed by the nonprofit TB Alliance, should be used in combination with bedaquiline and linezolid, with or without moxifloxacin (BPaL and BPaLM, respectively) [2]. Over the years, this regimen has been adopted in about 70 countries.

On August 9, 2024, India announced to roll out a new shorter, i.e., 26-week-long, BPaLM regimen comprising of four drugs: bedaquiline, pretomanid, linezolid, and moxifloxacin. The Health Ministry further detailed that the training for the health professionals will start by the end of August or first week of September at six training sites, each covering six states. The regimen has certain advantages, as mentioned in Table 1 [3,4].

**Table 1:** Advantages of the BPaLM regimen. BPaLM: bedaquiline, pretomanid, linezolid, and moxifloxacin References: [3,4]

BPaLM regimen	Advantages
	More effective
	Less pill burden
	Fewer adverse drug reactions
	Effective in preventing transmission
	Cost-effective compared to other regimens

This new regimen is based on the results obtained from a study coordinated by the Indian Council of Medical Research and the National Institute of Research in Tuberculosis, Chennai, which has reported more than 90 percent success rates. Moreover, the fatalities, which are commonly 14-17%, dropped to about 3-4% [3].

Similar results were reported in trials and studies across the world. A South African study reported a favorable outcome of 90%. Another multi-country ZeNIX study reported a 93% success rate [5].

In addition, this newer regimen is cost-effective, as the savings per patient treated in Pakistan, the Philippines, South Africa, and Ukraine are $746, $478, $757, and $2,636, respectively when shorter and longer regimens are substituted with BPaL/BPaLM regimens. With BPaL/BPaLM regimens, a greater number of patients would receive successful treatment; in the four nations, respectively, disability-adjusted life years prevented would be 20,179, 27,443, 33,384, and 21,924, and lives saved would be 411, 1,025, 1,371, and 829, respectively [4].

To conclude, the rollout of the newer BPaLM regimen is a great initiative by the Indian government, as India, which has nearly 27% of total TB cases globally, would benefit a lot from this rollout of the new regimen. This would also boost its efforts aimed at TB elimination by 2025 [3]. However, caution should be taken in this transition from the shorter and longer conventional regimens. Although comparatively low, several side effects related to the drugs involved in the BPaL regimens have been documented in the literature, and hence a timely monitoring and evaluation along with regular follow-ups for any adverse drug reactions would be imperative.
